# Development and User Experiences of a Biopsychosocial Interprofessional Online Course on Persistent Somatic Symptoms

**DOI:** 10.3389/fpsyt.2021.725546

**Published:** 2021-11-03

**Authors:** A. van Gils, L. M. Tak, H. Sattel, J. G. M. Rosmalen

**Affiliations:** ^1^University of Groningen, University Medical Center Groningen, Departments of Psychiatry and Internal Medicine, Groningen, Netherlands; ^2^Specialist Center for Persistent Somatic Symptoms & Somatic Symptom Disorders, Dimence Mental Health Care, Deventer, Netherlands; ^3^Department of Psychosomatic Medicine and Psychotherapy, The Technical University of Munich, Munich, Germany

**Keywords:** persistent somatic symptoms, interdisciplinary, eHealth, online course, education, biopsychosocial, somatic symptom disorder

## Abstract

**Background:** Communication between healthcare providers and patients with persistent somatic symptoms (PSS) is frequently hampered by mutual misunderstanding and dissatisfaction.

**Methods:** We developed an online, interprofessional course to teach healthcare providers the knowledge, skills, and attitude they need to diagnose and treat PSS in a patient-centered manner based on the biopsychosocial model. The course consisted of six modules of 45–60 min. Each module contained different types of assignments, based on six cases: videos, discussion boards, reading assignments, polls, and quizzes. For this study, we included (1) medical residents, following the course as part of their residency training, and (2) healthcare providers (general practitioners, medical specialists, physiotherapists, nurses, and psychologists), following the course as continuing vocational training. Throughout the course, participants were asked to fill out online surveys, enquiring about their learning gains and satisfaction with the course.

**Results:** The biopsychosocial approach was integrated across the modules and teached health care workers about recent insights on biological, psychological and social aspects of PSS. In total, 801 participants with a wide variety in clinical experience started the course; the largest groups of professionals were general practitioners (*N* = 400), physiotherapists (*N* = 124) and mental healthcare workers (*N* = 53). At the start of the course, 22% of the participants rated their level of knowledge on PSS as adequate. At the end of the course, 359 participants completed the evaluation questionnaires. Of this group, 81% rated their level of knowledge on PSS as adequate and 86% felt that following the course increased their competencies in communicating with patients with PSS (*N* = 359). On a scale from 1 to 10, participants gave the course a mean grade of 7.8 points. Accordingly, 85% stated that they would recommend the course to a colleague.

**Conclusion:** Our course developed in a co-design process involving multiple stakeholders can be implemented, is being used, and is positively evaluated by professionals across a variety of health care settings.

## Introduction

A substantial proportion of physical symptoms cannot be (fully) explained by a medical disease. This varies from ~20–35% in primary care to 30–50% in secondary care (1–4). Even though most physical symptoms are self-limiting, 10–30% of symptoms persist after a year, causing considerable suffering and disability (5). Those persisting somatic symptoms (PSS) are associated with increased use of healthcare resources and their medical costs rank among the highest of all patient groups (6). This is partly due to repeated referrals and investigations, which are often unhelpful and sometimes even cause iatrogenic damage (7).

PSS result from the complex interplay of biomedical, psychological and/or social (biopsychosocial) factors. This multifactorial etiology complicates the deduction of a clear diagnostic and treatment rationale used by all different types of health care providers (8).

Many healthcare providers perceive patients with PSS as “heartsink” or “difficult” (9). Many patients with PSS feel like they are not being taken seriously by healthcare providers (10). Misconceptions are found on both sides, hampering effective treatment and recovery of patients with PSS (11). For example, the labels that doctors use to describe PSS often lead patients to believe that the doctor is suggesting they are “putting on” or “imagining” their symptoms, or that they are “mad” (12). In addition, professionals from different disciplines use their own labels and concepts for PSS, often emphasizing either biomedical or psychosocial factors, leading to an inconsistent and suboptimal approach of patients with PSS. Also, doctors often feel pressurized and uncomfortable, because they feel patients demand (unnecessary) somatic interventions. However, research shows that it is mostly doctors proposing somatic interventions, not patients. If anything, patients with PSS seek for emotional support and reassurance (13). Clearing up these misconceptions calls for better interpersonal communication, a more patient-centered biopsychosocial approach across professionals from different disciplines and knowledge about treatment options. In recent years, a paradigm shift has emerged to organize care from a patient instead of a provider perspective. This means providing care that is respectful of and responsive to the needs, values, and preferences of individual patients, and actively involving patients in clinical decisions (14). Patient-centered care has many benefits: it improves job satisfaction among healthcare providers, patient wellbeing, treatment compliance, and health outcomes without increased use of healthcare resources (15). The treatment of PSS often involves somatic and psychosocial health care providers and requires a biopsychosocial approach and common interdisciplinary language.

To promote patient-centered care for patients with PSS, we aimed to develop an online course, teaching healthcare professionals from various disciplines the knowledge, skills, and attitude they need to adequately diagnose and treat PSS, based on a biopsychosocial perspective. Online learning (“e-learning”) is an innovative form of education, which is appreciated for its flexibility, convenience, and self-controlled learning pace (16). The use of different types of media and interactive tools increases motivation and promotes practically applied learning, resulting in more efficient learning experiences (17). In this paper, we describe the development of this course, healthcare provider satisfaction with the course, and self-reported effects on knowledge, skills, and attitude.

## Methods

### Course Development

PSS experts, educational experts, healthcare professionals from various disciplines (i.e., general practice, clinical psychology, psychiatry, physiotherapy, and various medical specialties), and a patient representative were involved in the development of the course. As a first step, workshops were organized to define the aim of the course, the intended target audience, relevant themes, and learning goals. Subsequently, we established a fixed structure for all course modules and decided on types of assignments that were to be used. Six cases were created for these assignments (see Box 1). Four of these cases were based on prototypical PSS patients, according to a focus group study amongst Dutch general practitioners (GPs) (the passive PSS patient, the anxious PSS patient, the distressed PSS patient, and the unhappy PSS patient) (18); The remaining two were created for a specific learning goal. then, all of the individual assignments were drafted, which included filming of interviews with experienced clinicians, recording “screencasts” (2–3 min explanatory videos), and filming re-enacted consultations with actors. The stakeholders were asked to give feedback on this first version of the course. Finally, a pilot was organized with 64 experienced GPs. Divided into two groups, they completed three course modules (1, 3, and 5 or 2, 4, and 6). Afterwards, structured focus group discussions were organized to gather qualitative feedback which was used to fine-tune the course in terms of form (structure, length of the modules, teaching methods) and content (topics, relevancy, level). The course was developed and piloted in the Dutch language and subsequently translated into English and German.

Box 1 **Cases of patients with PSS used in assignments throughout the course.**

***Case*** ***1***

*Forty one-year-old single mother of two visits her general practitioner (GP), because she is increasingly bothered by gastrointestinal complaints. She was diagnosed with irritable bowel syndrome 10 years ago, which runs in her family. The symptoms had been manageable for years, but recently she has been frequently experiencing diarrhea, flatulence, bloating, and fatigue. The patient feels very ashamed of these symptoms. She has no idea why the symptoms have worsened and does not know what to do about it*.

***Case 2***

*Since she has had the flu 6 months ago, a 19-year-old psychology student has been experiencing ongoing fatigue, headache, neck pain, and trouble concentrating. She regularly takes naps during the day, because she cannot stay awake. She is no longer able to play handball or study. She worries that her symptoms will not go away*.

***Case 3***

*A 43-year-old IT-specialist visits his GP, because he has been experiencing chest pains and palpitations for 2 weeks. Five months ago, he visited the emergency department with acute chest pain, which was classified as atypical, non-cardiac chest pain. The patient and his wife are very worried and insist they would like to be referred to a cardiologist. Two years ago, a friend died of a heart attack and the patient fears this might happen to him as well*.

***Case 4***

*Four months ago, a 32-year-old lawyer suffered from sudden and severe vertigo, nausea and vomiting. She was diagnosed with vestibular neuritis. The patient now visits her GP, because she keeps feeling dizzy and unsteady. She is also very tired and sometimes feels like she is “not quite there”. The patient feels stressed out, because the symptoms interfere with her demanding job*.

***Case 5***

*A 51-year-old man with type 2 diabetes has been suffering from generalized, chronic pain for 3 years. A rheumatologist could not find a medical explanation for the symptoms. The patient now visits his GP, because the pain in his hands and knees has increased. This has led him to cease his hobbies: fishing and playing cards with friends. The patient seems down. There is not much he enjoys in life*.

### Course Structure and Content

The aim of the course was to teach healthcare providers how to diagnose and treat PSS in a patient-centered manner. In order to facilitate interprofessional collaboration and communication between different types of healthcare providers using the biopsychosocial model as a basis, the course was designed for all healthcare providers involved in the care of patients with PSS, including GPS, medical specialists (internists, gastroenterologists, rheumatologists, gynecologists, neurologists, psychiatrists, rehabilitation specialists, and occupational physicians), physiotherapists, nurses, psychologists, and other mental healthcare workers.

### Participants

We recruited participants for this study in two ways. First, the online course was implemented in the training of medical residents from various specialties in the University Medical Centers of Groningen, Nijmegen and Amsterdam, the Netherlands. Second, the course was offered to various groups of Dutch healthcare providers as continuing vocational training, for which they had to pay € 100. In the Netherlands, registered healthcare providers are obliged to take a certain amount of accredited courses. Our course was accredited for GPs, medical specialists, physiotherapists, nurses, and psychologists. In order to recruit participants for this group, we promoted the course through a website (https://Grip.Health/Pages/Elearning), social media (twitter, linkedin), short articles in Dutch medical journals, and local/national meetings for healthcare providers. To be awarded accreditation points, these healthcare providers had to finish all of the course modules. Participants were recruited between September 2017 and June 2021.

### Evaluation

Throughout the course, participants were asked to fill out custom designed, integrated online surveys. These surveys were offered (1) before the start of the course (i.e., before the first course module), (2) after each of the course modules, and (3) after finishing the course (i.e., after the final course module).

#### Participant Characteristics

The survey before the start of the course contained questions on participants' sex, age, profession, and years of clinical experience.

#### Self-Reported Knowledge, Skills, and Attitude

The surveys before the start and after the end of the course contained general questions on participants' attitude toward and knowledge of PSS. The surveys at the end of the various course modules evaluated (improvements in) knowledge, skills, and attitude with regard to the specific themes of the module (i.e., whether learning goals were met). Items from all these surveys were phrased as statements with a five-point likert scale (fully disagree / disagree / neither disagree, nor agree / agree / fully agree). For the variables assessing learning goals of the individual modules, responses “agree” (4) and “fully agree” (5) were combined.

#### Satisfaction

The evaluative survey at the end of the course assessed participants' satisfaction with the course. Participants were asked to grade the course on a scale from 1 to 10. In the Netherlands this is a common scale in education, with six referring to pass, eight to good, and 10 to excellent. In addition, they were asked whether they would recommend the course to a colleague and whether the course content was directly applicable in their daily practice. These items were phrased as statements with a five-point Likert scale (fully disagree / disagree / neither disagree, nor agree / agree / fully agree). For these variables, responses “agree” (4) and “fully agree” (5) were combined.

## Results

### Development of the Course Modules

The workshop identified six themes with specific learning goals. The Canadian Medical Education Directives for Specialists (CanMEDS) framework (19) was used to link these themes (modules) and learning goals to relevant competencies for medical professionals. These CanMEDS competencies were then translated into six course modules (see Table 1). Each module had the exact same structure. It started with the learning goals of the module, followed by 6 to 15 short assignments (videos, discussion boards, reading assignments, polls, and quizzes). All modules ended with a take-home-message, an evaluative survey and a “further reading” segment.

**Table 1:** Learning goals per course module with relevant CanMEDS competencies.

**Module**		**Learning Goals**	**CanMEDS competency^a^**						
		***After following this module, the participant will:***	**a**	**b**	**c**	**d**	**e**	**f**	**g**
1	Introduction	• Be more aware of their attitude toward patients with PSS	.	.	.	.	.	.	√
		• Have gained insight into 10 common misconceptions about PSS							
2	Basic knowledge	• Have gained basic knowledge on the terminology, prevalence, prognosis, and etiology of PSS	√	.	.	.	.	√	√
3	Assessment	• Be able to make informed decisions on diagnostic testing, avoiding unnecessary procedures	√	.	.	.	√	√	.
		• Know how to minimize the chance of misdiagnosis							
		• Be able to recognize and explore the 5 symptom dimensions (physical, cognitive, emotional, behavioral, and social)							
		• Be able to recognize psychiatric comorbidity							
4	Consultation	• Be able to recognize signs that a patient feels unheard Know how to use physical examination to effectively reassure a patient	√	√	.	.	√	.	√
		• Be able to explain the working diagnosis “PSS” to a patient							
		• Be able to recognize and prevent a common negative interaction pattern							
5	Treatment in primary care	• Be able to assess the severity of PSS	√	√	√	√	.	.	.
		• Know methods to motivate patients for behavior change							
		• Be able to set treatment goals together with a patient and monitor progress							
6	Collaboration	• Know how to improve communication and collaboration with other health care providers	√	√	√	√	.	.	√
		• Know which are key elements in a good (referral) letter							
		• Know when and how to refer a patients with PSS to mental healthcare							

a *Canadian Medical Education Directives for Specialists (CanMEDS) is a framework, aimed to improve care by enhancing physician training, including the following competencies/roles (19) a, medical expert; b, communicator; c, collaborator; d, leader; e, health advocate; f, scholar; g, professional*.

The biopsychosocial approach was integrated across the modules and teached health care workers about recent insights on biological, psychological and social aspects of PSS. In module 1, the main theme was correcting the misconceptions related to PSS being a problem (only) with a psychological origin. Module 2 introduced the etiology of PSS, discussing the contribution of biological, psychological and social factors. Module 3 included information on diagnoses of somatic and psychiatric diseases, and on how to explore physical, cognitive, emotional, behavioral and social symptom dimensions. Module 4 focused on relational aspects and communication during consultations, with exercises about biopsychosocial explanations for PSS. In module 5, treatment was introduced, with information on optimal communication for motivation of patients. Finally, module 6 focused on interprofessional collaboration and role differentiation, how to work as a team, and educated participants on how to communicate with healthcare professionals from different disciplines and when to refer a patient to mental health care.

### Participants

Before the start of the course, 801 participants filled out the general survey (see Table 2). Most of these were GPs, physiotherapists and psychologists or other mental health care providers (including residents and trainees). Years of clinical experience ranged from 0 to 45 and a median of 6 years [interquartile range (IQR) (3–20)]. Of the 801 participants, 22% rated their level of knowledge on PSS as adequate, and only 14% of participants indicated that they did *not* find patients with PSS difficult to deal with. Of all participants, 91% stated that they considered PSS a serious health problem and 50% indicated they had a special interest in PSS.

**Table 2:** General characteristics of online PSS course participants (*N* = 801).

**Variable**	
Male sex, *n* (%)	213 (26.6%)
Age in years, median (IQR)	33 (29–49)
Clinical experience in years, median (IQR)	6 (3–20)
Profession, including residents and trainees, *n* (%)	
General practitioner	400 (49.9%)
Physiotherapist	124 (15.5%)
Psychologist or other mental health worker	43 (5.4%)
Psychiatrist	10 (1.2%)
Internist, rheumatologist, gastroenterologist	34 (4.2%)
Rehabilitation specialist	15 (1.9%)
Neurologist	6 (0.7%)
Other	98 (12.2%)
Unknown (not reported)	47 (5.9%)

Participants generally rated their (improvements in) knowledge, skills, and attitude regarding the specific learning goals of the six modules as satisfactory, with at least 70% reporting improvements (Table 3). Exceptions are a change in attitude, a learning goal of the first module, that was only reported by 33%. In addition, the improvement in knowledge on motivating patients for behavior change (module 5) was reported by 59%, and knowledge on when and how to refer patients to mental healthcare in 55 and 66%, respectively (module 6).

**Table 3:** Self-rated knowledge, skills, and attitude on PSS after each of the course modules.

**Learning gains after course module**	**Module**	**(Fully) agree**	**N**
Increased awareness of attitude toward PSS	1	73%	680
Changed attitude toward PSS	1	33%	680
Knowledge on terminology adequate	2	80%	516
Knowledge on prevalence and prognosis adequate	2	79%	516
Knowledge on etiology adequate	2	83%	516
Increased awareness of consequences diagnostic procedures and referral	3	74%	447
Better able to recognize and explore symptom dimensions	3	72%	447
Improved ability to recognize when patient feels unheard	4	80%	400
Knows how to explain working diagnosis PSS to patients	4	83%	400
Knows how to formulate treatment goals together with patients and how to monitor progress	5	75%	381
Knows better how to motivate patients for behavior change	5	59%	381
Changed writing of letters about patients with PSS	6	83%	347
Knows when to refer a patient with PSS to mental healthcare	6	55%	347
Knows how to refer a patient with PSS to mental healthcare	6	66%	347

### Self-Rated Knowledge, Skills, and Attitude on PSS

**Figure 1:**
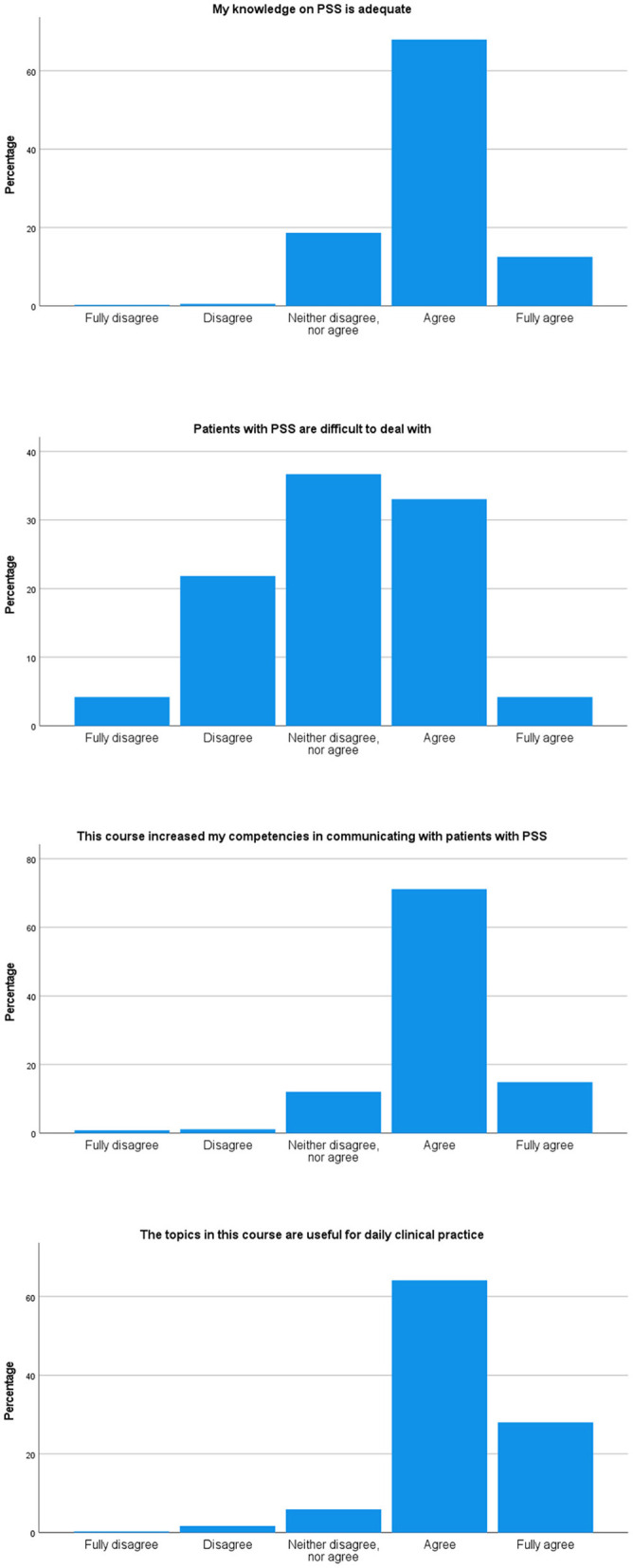
Self-rated knowledge, skills, and attitude on PSS at the end of the course (*N* = 359).

At the end of the course, 359 participants filled out the evaluative survey. After taking the course, 81% of participants rated their level of knowledge on PSS as adequate, and 86% felt that following the course increased their competencies in communicating with patients with PSS (see Figure 1). Of the participants who completed the full course, the range of time spend was 2–30 h (mean 7.8 h, mode 6 h).

### Satisfaction

When asked to grade the course, participants gave the course an overall mean score of 7.8 (SD 0.9, minimum-maximum 1–10). Accordingly, 85% would recommend the course to a colleague and 92% found that what they had learned during the course could be directly applied in their daily practice.

## Discussion

In this study, we explored user experiences with an online, interprofessional course on PSS based on the biopsychosocial model. Our course developed in a co-design process involving multiple stakeholders can be implemented, is being used, and is positively evaluated by professionals across a variety of health care settings.

Our study confirms the findings of previous studies about the perspective of healthcare providers on patients with PSS and their ability to manage these patients. Our baseline survey shows that only 14% of participants did *not* find patients with PSS hard to deal with. This is in line with several previous studies, showing that physicians perceive patients with PSS as difficult, especially when they present with multiple symptoms (20, 21). In addition, 22% of our study participants rated their knowledge on PSS as adequate before taking the course. A previous survey amongst physicians also shows that a substantial proportion perceive themselves as insufficiently competent in managing patients with PSS (22). These findings highlight the need for education and training on PSS.

With regard to learning gains, participants generally rated their (improvements in) knowledge, skills, and attitude as satisfactory. Even though 73% indicated that the course had increased their awareness of their attitude toward PSS, only 33% reported that their attitude had actually changed. However, this is not necessarily a problem. At the start of the course, 91% of participants stated that they considered PSS a serious health problem, which suggests that these participants might already have had a positive attitude. Furthermore, a limited number of participants reported that they knew when (55%) and how (66%) to refer patients with PSS to mental healthcare. We therefore conclude this course topic needs revision and extra attention.

Participants who filled out the survey at the end of the course were satisfied with the course. This is in line with previous studies on e-learning in medical and nonmedical fields, which have consistently demonstrated high satisfaction rates (23). Although we did not enquire appreciation for different aspects of the course (form, content, etc.), our findings indicate that e-learning is an appreciated form of education on the topic of PSS. This fits with a large body of literature pointing out the advantages of online learning, which include its flexibility, convenience, and self-controlled learning pace (16, 17, 24).

A strength of this study is the broad spectrum of healthcare providers included in the study. The course was developed with the help of many relevant stakeholders (PSS experts, education experts, healthcare professionals from different disciplines, and a patient representative), in order to be suitable for a large variety of healthcare providers. The group of participants represents the full spectrum of healthcare professionals from these different disciplines.

The most important limitation of the current study is the occurrence of several types of bias. First, self-selection by healthcare providers probably led to a selection bias. At baseline, 50% of participants indicated they had a special interest in PSS. This affinity might have increased their appreciation of the course content. On the other hand, some topics may have already been known and therefore considered redundant.

Secondly, attrition bias arose as a consequence of the manner of data collection. Participants were requested to (voluntarily) fill out several surveys, yet not all of them filled out all of the surveys. A large difference can be observed between the number of participants, who filled out the survey at the start of the course (*N* = 801), and the number of participants, who filled out the survey at the end of the course (*N* = 359). This might have influenced our results, since especially motivated participants might have completed the evaluation, and thus data are missing non at random (25). Apart from creating a bias in study results, attrition is a more general issue in e-learning, which requires more motivation and self-discipline than traditional teaching methods, such as lectures or workshops (16). Another limitation of this study is the lack of standardized, validated instruments to assess satisfaction and learning gains. A final limitation is our data collection in a real-world implementation setting. The evaluations were included in the e-learning, and it was not possible to couple the evaluations of the different modules due to the lack of a personal identifier in the data extracted from the learning management system. Therefore, we were not able to link evaluation data to personal characteristics and make statistical inferences (for instance, characteristics of completers / non-completers). This also implies that the data obtained before and after the training cannot be directly compared, since this would require an analysis on the sample that filled in both evaluations.

The current study explored learning gains through self-assessment by healthcare providers. Because the course aimed to improve patient-centered care, it would be interesting to study patients' perspectives of their healthcare providers' communication skills and attitude in the future. Another way to gain a more objective impression of improvements in knowledge, skills, and attitude, would be to let observers rate consultations before and after healthcare providers have taken the course.

The development of the course and conduction of the pilot study took place in the Netherlands. Thereafter, the course was translated into English and German, which allows the course to be used, studied, and further developed internationally. In addition, we are developing extra course modules with specific themes, such as PSS in children, mental health care for PSS and sex- and gender-sensitive care for PSS. The course could be further improved by involving stakeholders from the social domain, such as social workers. Accreditation of this course by the professional organization of social workers could improve the knowledge in skills in these professionals, and help to address the social aspects of the biopsychosocial model in patients with PSS.

In conclusion, according to healthcare providers, this online, interprofessional course is an effective and satisfying way to learn about PSS. Observer- and patient-rated outcomes are to be studied in the future.

## Author's Note

Neither the manuscript nor any significant part of it is under consideration for publication elsewhere. A pilot study-based on 119/60 participants instead of 801/359 participants in the current study-was published as part of a PhD thesis (https://research.rug.nl/nl/publications/developing-e-health-applications-to-promote-a-patient-centered-ap) but has not been submitted to a scientific journal or peer-reviewed previously. All authors listed have contributed significantly to the manuscript and consent to their names on the manuscript. In addition, all authors consent to publication.

## Data Availability Statement

The raw data supporting the conclusions of this article will be made available by the authors, without undue reservation.

## Ethics Statement

Ethical review and approval was not required for the study on human participants in accordance with the local legislation and institutional requirements. The patients/participants provided their written informed consent to participate in this study.

## Author Contributions

All authors listed have made a substantial, direct and intellectual contribution to the work, and approved it for publication.

## Funding

This study was financially supported by a grant from EIT Health (Grant Number: 681282).

## Conflict of Interest

The authors declare that the research was conducted in the absence of any commercial or financial relationships that could be construed as a potential conflict of interest.

## Publisher's Note

All claims expressed in this article are solely those of the authors and do not necessarily represent those of their affiliated organizations, or those of the publisher, the editors and the reviewers. Any product that may be evaluated in this article, or claim that may be made by its manufacturer, is not guaranteed or endorsed by the publisher.
